# Dianhydrogalactitol synergizes with topoisomerase poisons to overcome DNA repair activity in tumor cells

**DOI:** 10.1038/s41419-020-02780-8

**Published:** 2020-07-24

**Authors:** Beibei Zhai, Yue Li, Sudha Sravanti Kotapalli, Jeffrey Bacha, Dennis Brown, Anne Steinø, Mads Daugaard

**Affiliations:** 1 Vancouver Prostate Centre, Vancouver, BC V6H 3Z6 Canada; 2 Department of Urologic Sciences, University of British Columbia, Vancouver, BC V5Z 1M9 Canada; 3 Interdisciplinary Oncology Program, University of British Columbia, Vancouver, BC V5Z 4S6 Canada; 4 Formerly affiliated with DelMar Pharmaceuticals, Inc., Vancouver, Canada; 5 ORP Canada Ltd., Vancouver, BC V6J 2J1 Canada; 6 DelMar Pharmaceuticals (BC) Ltd., Vancouver, BC V5Z 1K5 Canada; 7 DelMar Pharmaceuticals, Inc., Menlo Park, CA 94025 USA

**Keywords:** Chemotherapy, DNA damage and repair

## Abstract

1,2:5,6-Dianhydrogalactitol (DAG) is a bi-functional DNA-targeting agent currently in phase II clinical trial for treatment of temozolomide-resistant glioblastoma (GBM). In the present study, we investigated the cytotoxic activity of DAG alone or in combination with common chemotherapy agents in GBM and prostate cancer (PCa) cells, and determined the impact of DNA repair pathways on DAG-induced cytotoxicity. We found that DAG produced replication-dependent DNA lesions decorated with RPA32, RAD51, and γH2AX foci. DAG-induced cytotoxicity was unaffected by MLH1, MSH2, and DNA-PK expression, but was enhanced by knockdown of BRCA1. Acting in S phase, DAG displayed selective synergy with topoisomerase I (camptothecin and irinotecan) and topoisomerase II (etoposide) poisons in GBM, PCa, and lung cancer cells with no synergy observed for docetaxel. Importantly, DAG combined with irinotecan treatment enhanced tumor responses and prolonged survival of tumor-bearing mice. This work provides mechanistic insight into DAG cytotoxicity in GBM and PCa cells and offers a rational for exploring combination regimens with topoisomerase I/II poisons in future clinical trials.

## Introduction

Glioblastoma multiforme (GBM) is the most common and aggressive primary malignant brain tumor in adults^1^. Current standard-of-care for newly diagnosed GBM patients include surgical resection, concomitant radiation with alkylating agent temozolomide (TMZ), and adjuvant TMZ therapy afterwards^2^. Although these treatments improve overall survival, nearly all patients experience TMZ resistance and tumor recurrence, and therefore the prognosis of GBM patients remains poor with a median survival time of 12–15 months^2,3^. 1,2:5,6-Dianhydrogalactitol (DAG) is a bi-functional DNA-targeting agent causing N^7^-guanine alkylation and inter-strand crosslinks^4^. It is a small water-soluble agent that readily crosses blood–brain-barrier^5^. Recently, DAG has been tested and shown activity either alone or in combination with radiation therapy in GBM clinical trials^6^. However, a detailed understanding of DAG cytotoxicity and cellular survival mechanisms is needed in order to further improve the outcomes of GBM therapeutic strategies in clinic.

Prostate cancer (PCa) is the most frequently diagnosed cancer in males in North America, with one out of six men developing PCa at some point during his lifetime. PCa is the second leading cause of malignancy-related mortality in men, following lung cancer^7^. The treatment strategies for PCa vary depending on cancer type and grade, metastasis status, patient’s age, and prior treatments. Local prostate-confined disease is normally managed by active surveillance, surgery, and radiation therapy. For advanced stage or metastatic PCa, androgen deprivation therapy is commonly included in addition to radiotherapy^8^. However, after a median remission for 14–30 months, the majority of the high-risk patients who receive androgen deprivation therapy progress with castration-resistant prostate cancer (CRPC)^8–10^. At present, taxane (docetaxel) plus prednisone is the first-line chemotherapy for CRPC patients, but almost all CRPC patients eventually relapse during or soon after taxane-based chemotherapy^11,12^. Therefore, novel treatment strategies are urgently needed to overcome resistance challenges and improve the survival of CRPC patients.

Topoisomerases are nuclear enzymes that control DNA supercoiling and entanglement, and they play essential roles in DNA replication, transcription, and genome stability in cells^13^. There are two types of DNA topoisomerases. Topoisomerase I generates single-strand breaks in DNA and topoisomerase II cuts both DNA strands in order to relax the supercoils and fix topological problems in chromatin. After the cutting and untangling, these enzymes re-ligate the cleaved strands to re-establish the intact DNA structure^14,15^.

Topoisomerase poisons are a distinct class of chemotherapeutic agents. They block the ligation function of the topoisomerases, leading to formation of stable topoisomerase-cleaved DNA complexes with single- and double-strand breaks (DSBs) that eventually triggers apoptosis and other types of cell death^15,16^. Topoisomerase poisons are commonly used in the treatment of solid tumors and show various efficacies depending on tumor types^17–20^. The topoisomerase II poison, mitoxantrone, was approved by FDA in 1996 for the treatment of PCa and some studies show clinical benefits of topoisomerase II poison etoposide in combination regimens for the treatment of patients with CRPC^15,21,22^. Recent clinical trials using the topoisomerase I poison irinotecan in combination with bevacizumab and/or TMZ in the treatment of recurrent GBM patients demonstrate moderate effectiveness and tolerance^23,24^.

In the present study, we interrogated DNA damage response pathways activated in GBM and PCa cells after treatment of DAG with the aim of uncovering potential combination treatment opportunities. Informed by this analysis, we subsequently investigated the cytotoxic activity of DAG in combination with topoisomerase I poisons (camptothecin and irinotecan), a topoisomerase II poison (etoposide), and a microtubule depolymerization inhibitor (docetaxel).

## Results

### DAG shows broad cytotoxicity in GBM and PCa cell lines

**Fig. 1:**
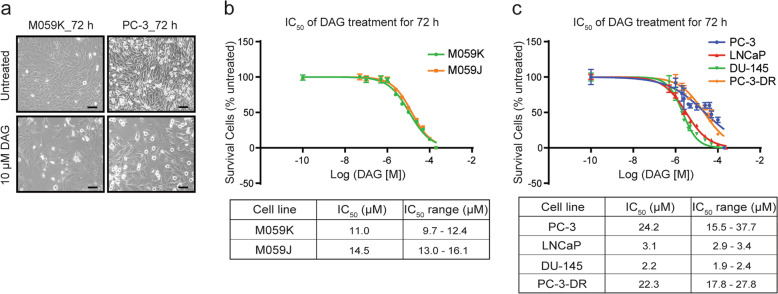
Cytotoxic effect of DAG in GBM and PCa cell lines. **a** M059K and PC-3 cells were treated with or without 10 μM DAG in complete medium for 72 h. The representative bright-field images were shown and the scale bar represents 100 μm. **b**, **c** Two GBM (M059K and M059J) and four PCa (PC-3, LNCaP, DU-145, and PC-3-DR) cell lines were treated with different concentrations of DAG (0, 50 nM, 100 nM, 500 nM, 1 μM, 2.5 μM, 5 μM, 10 μM, 25 μM, 50 μM, 100 μM, and 200 μM) for 72 h in 96-well culture plates. Following the treatment, crystal violet assay was performed and the IC_50_ values of DAG for each cell line were calculated by fitting a sigmoidal dose-response curve to the data using GraphPad Prism 6. The data on the curve are presented as mean ± standard deviation. Three individual experiments were performed for each cell line.

DAG has previously been shown to have cytotoxic effects in lung cancer cells^25^. To investigate if DAG exerts a similar cytotoxic activity in GBM and PCa cells, we tested DAG cytotoxicity and determined half-maximal inhibitory concentration (IC_50_) values in two GBM and four PCa cell lines, among which PC-3-DR is resistant to docetaxel^26^. Treatment of both GBM and PCa cells with 10 μM DAG for 72 h caused visible morphological changes and decreased cell density (Fig. 1a). A cell viability analysis with different concentrations of DAG treatment for 72 h showed concentration-dependent decreased survival in all cell lines with corresponding IC_50_ values in the low µm range (Fig. 1b, c). In summary, these data demonstrate cytotoxic activity of DAG in both GBM and PCa cell lines.

### DAG causes replication-dependent DNA damage

**Fig. 2:**
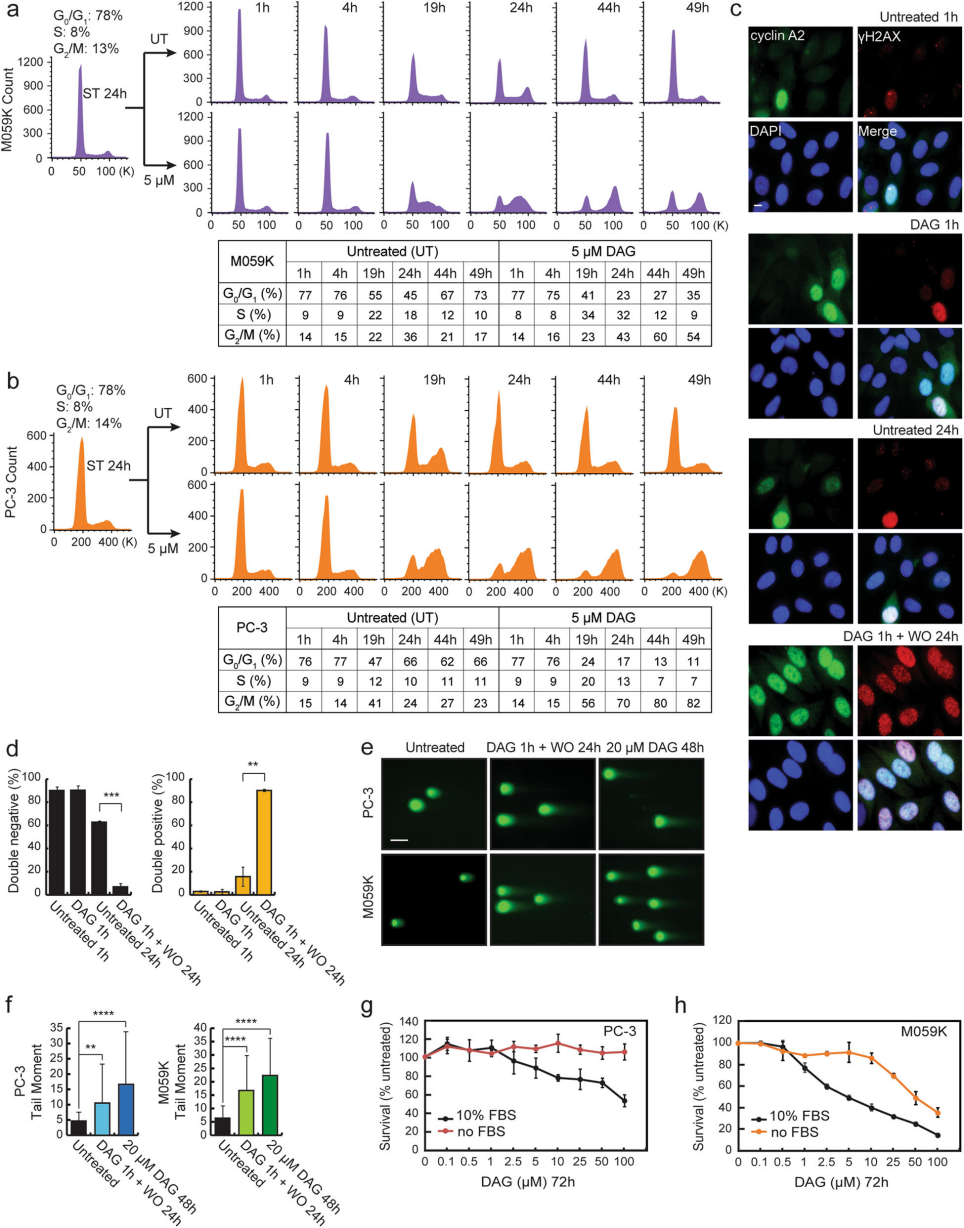
DAG treatment causes S/G_2_ phase cell cycle arrest and induces replication-dependent DNA damage. **a**, **b** Serum-starved (ST for 24 h) M059K and PC-3 cells were treated with or without 5 μM DAG in complete medium for the indicated periods of time. Then the cells were collected for cell cycle analysis using PI staining as described in “Materials and Methods”. The representative flow cytometric histograms from two individual experiments are shown with the corresponding percentages of cell populations at G_0_/G_1_, S, and G_2_/M phases. **c** PC-3 cells were synchronized at G_0_/G_1_ phase by 24 h serum starvation before treatment with 50 μM DAG for 1 h in complete medium. After the treatment, DAG was removed and the cells were continued to incubate in complete medium for another 24 h (DAG 1 h + WO 24 h). WO stands for washout. After that, cells were fixed, permeabilized, and immunostained with anti-cyclin A2 (green) and anti-ɣH2AX (red) antibodies. Representative IF images are shown from two independent experiments. The scale bar stands for 10 μm. **d** From each experimental condition in **c**, we examined 100–120 cells and calculated the percentages of cyclin A2-/ɣH2AX- (double negative) and cyclin A2 + /ɣH2AX + (double positive) populations of cells. The corresponding statistical analysis shows significance (**p ≤ 0.01; ***p ≤ 0.001). **e** PC-3 and M059K cells were treated with 20 μM DAG for 48 h or 50 μM DAG for 1 h (in quiescent cells by 24 h serum starvation) followed by 24 h incubation without the drug in complete medium (DAG 1 h + WO 24 h). DSBs were analyzed by neutral comet assay and representative images are shown. The scale bar stands for 50 μm. **f** Tail moment from 50–100 cells at each experimental condition in **e** was analyzed by ImageJ (OpenComet) software and shows significance compare to untreated cells (**p ≤ 0.01; ****p ≤ 0.0001). **g**, **h** PC-3 or M059K cells were seeded in 96-well plates and cultured for 24 h in either serum-deprived medium or complete medium. Then the cells were treated with different concentrations of DAG for 72 h followed by crystal violet assay. Cell survival rates compared to untreated condition were shown as mean ± standard deviation.

We next examined cell cycle progression after DAG treatment using flow cytometry in propidium iodide (PI)-stained M059K and PC-3 cells. The cells were first synchronized in G_0_/G_1_ cell cycle phase by a 24 h serum starvation block. After that, the cells were released from the G_0_/G_1_ block by addition of complete medium with or without 5 μM DAG, followed by flow cytometric analysis. Compared with untreated cells that progressed with a normal cell cycle profile after culturing in complete medium, DAG-treated cells displayed a strong time-dependent S/G_2_-phase cell cycle arrest starting at 19 h post serum addition (Fig. 2a, b), indicating that DAG-mediated cytotoxicity depends on DNA replication in these cells. As cyclin A is exclusively expressed in S and G_2_ cell cycle phases^27^ and phospho-histone H2AX (ɣH2AX) is extensively used as a surrogate marker for DNA DSBs^28^, we next examined cyclin A2 and ɣH2AX expression using immunofluorescent (IF) staining in serum-starved PC-3 cells with or without DAG pulse treatment (50 μM DAG for 1 h) in complete medium. The PC-3 cells demonstrated intensive cyclin A2 (green) and ɣH2AX (red) expression after DAG pulse treatment followed by 24 h recovery without the drug (DAG 1 h + WO 24 h) (Fig. 2c). The cyclin A2+/ɣH2AX+ cell populations were significantly increased after DAG treatment followed by 24 h washout (DAG 1 h + WO 24 h) (Fig. 2d), indicating that DAG-induced DNA DSBs at S/G_2_ cell cycle phases in these cells. This was confirmed by a neutral comet assay, which detected significant amounts of DSBs occurring after DAG pulse treatment followed by 24 h washout or continuous treatment for 48 h in both PC-3 and M059K cells (Fig. 2e, f; Supplementary Fig. S1). To further substantiate our observation, a cell survival analysis was performed using different concentrations of DAG in the culture medium with or without serum. In both PC-3 and M059K cells, serum deprivation, which prohibited the cells from entering S phase, rescued the cells from DAG-induced cytotoxicity (Fig. 2g, h). All together, these data confirm that DAG-induced DNA inter-strand crosslinks lead to replication-dependent DNA damage in cancer cells.

### DAG cytotoxicity is affected by homologous recombination DNA repair activity

In eukaryotic cells, there are two major pathways that facilitate repair of DSBs in DNA, which are non-homologous end-joining (NHEJ) and homologous recombination (HR). DNA-dependent protein kinase (DNA-PK), a nuclear serine/threonine protein kinase, is essential for NHEJ repair activity^29^. DNA-PK is composed of two predominant components, a ring-shaped Ku 70/80 heterodimer and a 460-kD DNA-dependent protein kinase catalytic subunit (DNA-PK_CS_)^30^. Two human GBM cell lines, M059K and M059J, derived from the same tumor specimen show different DNA-PK activity. Although M059K cells express normal levels of DNA-PK, are NHEJ proficient and are resistant to radiation, M059J cells lack DNA-PK, are NHEJ deficient, and more sensitive to radiation^31,32^. These two cell lines are therefore commonly used as a model system for investigating NHEJ involvement^33–35^. To investigate whether the NHEJ pathway might contribute in repairing DAG-induced DNA DSBs, we evaluated the cytotoxic effect of DAG in M059K and M059J cells. Cell survival was comparable between these two cell lines (Fig. 1b), suggesting that the NHEJ pathway is not essential for coping with DAG-induced DNA lesions.

**Fig. 3:**
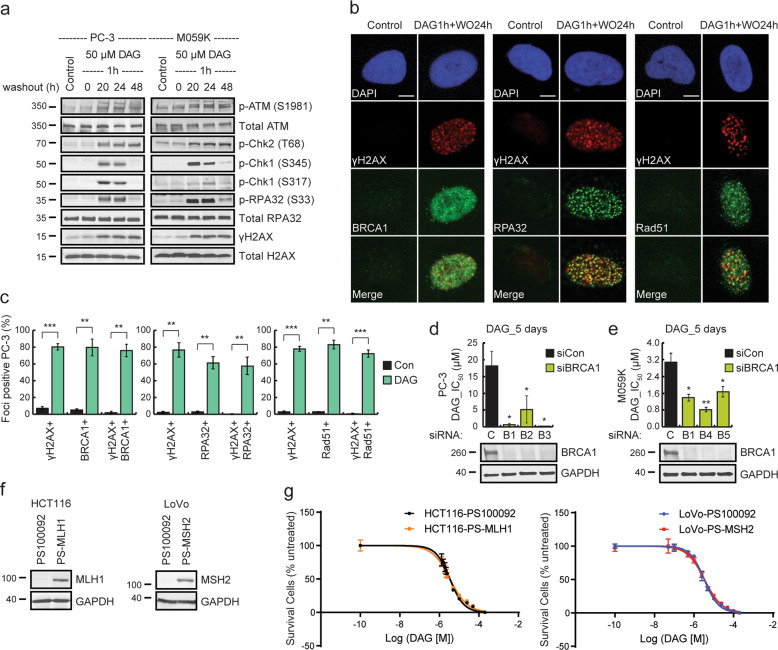
DAG-induced DNA damage activates HR pathway. **a** PC-3 and M059K cells were synchronized at G_0_/G_1_ phase by serum starvation for 24 h. The quiescent cells were treated with 50 μM DAG for 1 h followed by washout and additional incubation of 0, 20, 24, or 48 h in complete medium. Cell lysates were then analyzed for HR mediators by western blot from three independent experiments. **b** Quiescent PC-3 cells were treated with 50 μM DAG for 1 h followed by incubation without the drug for 24 h. Cells were pre-extracted by cytoskeletal buffer, fixed, permeabilized, and probed with corresponding antibodies for indicated proteins. Representative confocal images are shown from three independent experiments. The scale bar represents 5 μm. **c** PC-3 (80–150 cells) from each condition in **b** were quantified for foci-positive cells with statistical analysis (**p ≤ 0.01; ***p ≤ 0.001). **d**, **e** PC-3 or M059K cells were knocked down of BRCA1 by transient transfection using non-overlapping BRCA1-targeting siRNAs (B1, siBRCA1–2; B2, siBRCA1–14; B3, siBRCA1–25; B4, siBRCA1–15; or B5, siBRCA1–17). The negative control siRNA (C) was also included. After 24 h transfection, the cells were treated with different concentrations of DAG (0, 100 nM, 500 nM, 1 μM, 1.5 μM, 2.5 μM, 5 μM, 10 μM, 25 μM, 50 μM, 100 μM, and 200 μM) for 5 days followed by crystal violet assay. The IC_50_ values of DAG in control or BRCA1 knockdown cells were calculated using GraphPad Prism 6. The data are presented as mean ± standard deviation from three independent experiments with statistical significance (*p ≤ 0.05; **p ≤ 0.01). In parallel western blot analysis confirmed BRCA1 knockdown. **f** Cell lysates were extracted from two pairs of isogenic colorectal cancer cell lines. Western blot analysis showed the expression of MLH1 and MSH2 in HCT116-PS-MLH1 and LoVo-PS-MSH2 cells, respectively, from three independent experiments. **g** The two pairs of isogenic colorectal cancer cells were treated with different concentrations of DAG for 3 days followed by crystal violet assay. The data in the curve are presented as mean ± standard deviation from three independent experiments.

In lung cancer, DAG-induced DNA DSBs are predominantly repaired by the HR pathway^25^. To determine whether HR is also active in repair of DAG-induced DNA damage in GBM and PCa cells, we preformed western blot analysis to check the DNA DSB sensors and effectors involved in the HR pathway after DAG treatment in both PC-3 and M059K cells. Consistently, DAG pulse treatment followed by 20–24 h recovery in complete medium, induced phosphorylation of the key HR mediators, including ataxia telangiectasia mutated kinase (ATM), Chk1, Chk2, and RPA32 in both PC-3 and M059K cells (Fig. 3a)^36–39^. The increased ɣH2AX expression again confirmed the presence of DNA DSBs upon DAG treatment (Fig. 3a). To further confirm this mechanism, PC-3 cells were examined for the recruitment of major HR repair proteins including BRCA1, RPA32, and Rad51^39^ to ɣH2AX foci induced by DAG treatment followed by 24 h recovery without the drug exposure using confocal microscopy (Fig. 3b). Colocalization was detected with statistical significance between BRCA1, RPA32, Rad51, and ɣH2AX foci (Fig. 3c). As our data indicated that the HR repair pathway was involved in the DNA damage response to DAG, we predicted that GBM and PCa cells deficient in HR would be hyper-sensitive to DAG treatment. To investigate this, we knocked down BRCA1, a critical component in the HR pathway^40,41^, and reassessed sensitivity to DAG. BRCA1 knockdown in both PC-3 and M059K cells using three non-overlapping siRNAs significantly increased the sensitivity to DAG treatment (Fig. 3d, e). These results suggest that HR is involved in resolving DAG-induced DNA DSBs in GBM and PCa cells.

TMZ is commonly used as the first-line chemotherapy for GBM patients^2,42^, but resistance to TMZ is still a critical challenge in the clinic. TMZ is a monofunctional alkylating agent that induces DNA damage through O^6^-methylguanine^43^. This type of DNA lesions is repaired by O^6^-methylguanine-DNA-methyltransferase (MGMT), which is the primary mechanism for TMZ resistance in GBM treatment affecting majority of GBM patients^44^. When MGMT is not present in GBM cells, O^6^-methylguanine-triggered base mispairing activates mismatch repair (MMR) pathway. However, repetitive failure of repair by MMR upon TMZ-alkylation leads to DNA strand breaks and eventually cell death^45–47^. Therefore, in MGMT-negative tumors, MMR deficiency becomes a secondary mechanism for TMZ resistance in GBM treatment^47,48^. Moreover, MMR deficiency is also reported to mediate platinum-resistance in the treatment of many other malignant diseases, such as lung, bladder, colorectal, and ovarian cancers^49^. Previous studies have demonstrated that DAG treatment is effective in both MGMT positive and negative GBM cells, suggesting that DAG may be able to circumvent MGMT-mediated TMZ resistance^6^. Eukaryotic cells contain several proteins responsible for the MMR activity, among which MLH1 and MSH2 proteins are the key components for different heterodimers (MLH1-PMS2, MLH1-PMS1, MLH1-MLH3; MSH2-MSH6, MSH2-MSH3) that dictate substrate specificity and cellular function^50–53^. To investigate if DAG can circumvent the secondary mechanism of TMZ resistance, MMR, we generated MLH1-deficient HCT116-PS100092 and MSH2-deficient LoVo-PS100092 cells together with their isogenic MLH1-proficient HCT116-PS-MLH1 and MSH2-proficient LoVo-PS-MSH2 cells by lentivirus transduction of the corresponding MLH1 and MSH2 genes, respectively. Western blot analysis confirmed the expression of MLH1 and MSH2 in HCT116-PS-MLH1 and LoVo-PS-MSH2 cells (Fig. 3f). Treatment with DAG for 72 h induced comparable toxicity in these two pairs of isogenic cell lines (Fig. 3g), suggesting that MLH1 and MSH2 were not particularly important in the repair of DAG-induced DNA lesions. All together, these data support the idea that HR is the predominant DNA repair mechanism activated by DAG-induced inter-strand crosslinks and promote DAG as a candidate chemotherapy for the treatment of patients with HR deficiency.

### DAG synergizes with topoisomerase poisons in vitro and enhances irinotecan efficacy in vivo

The DAG-induced cytotoxicity in S/G_2_ phase of the cell cycle hints that DAG might synergize with other mechanistically non-overlapping S-phase-targeting agents such as topoisomerase poisons. To investigate this, we treated cancer cells with DAG in combination of etoposide, irinotecan, or camptothecin. Combination index (CI) values were determined to assess whether the effect of each combination was synergistic or additive according to Chou–Talalay method^54^. CI values of < 1 indicate synergism; CI values = 1 represent additive effect; and CI values of > 1 indicate antagonism^54^. DAG and etoposide combination treatment at the indicated molar ratios showed CI values at ED60, ED75, and ED90 of 0.81, 0.62, and 0.42, respectively, in M059K cells; and CI values at ED50, ED75, and ED90 of 0.58, 0.48, and 0.42, respectively, in PC-3 cells (Tables 1, 2). DAG and irinotecan combination treatment at the indicated molar ratios showed CI values at ED50, ED75, and ED90 of 0.78, 0.63, and 0.53, respectively, in M059K cells; and CI values at ED75, ED85, and ED90 of 0.78, 0.59, and 0.49, respectively, in PC-3 cells (Tables 1, 2). DAG and camptothecin combination at the indicated molar ratios showed CI values at ED75, ED90, and ED95 of 0.68, 0.59, and 0.54, respectively, in PC-3 cells (Table 2). Moreover, we also tested combination treatment of DAG with etoposide or camptothecin in A549, a lung cancer cell line we used in our previous study. Indeed, these combinations in A549 also showed synergistic effects with CI values of <1 at indicated cytotoxic effect (Fa) levels (Table 3).

**Table 1:** Combination treatments in M059K GBM cell line.

DAG + etoposide^a^		DAG + irinotecan^b^		DAG + docetaxel^c^	
Cytotoxic effect (Fa)	Combination index (CI)	Cytotoxic effect (Fa)	Combination index (CI)	Cytotoxic effect (Fa)	Combination index (CI)
ED60	0.81 ± 0.12	ED50	0.78 ± 0.09	ED50	3.07 ± 1.08
ED75	0.62 ± 0.11	ED75	0.63 ± 0.09	ED75	3.73 ± 1.35
ED90	0.42 ± 0.09	ED90	0.53 ± 0.10	ED90	4.57 ± 1.70

^a^Molar ratio of DAG:etoposide is 2.5:1.

^b^Molar ratio of DAG:irinotecan is 3.4:1.

^c^Molar ratio of DAG (µM):docetaxel (nM) is 1.3:1 in M059K cell line.

**Table 2:** Combination treatments in PC-3 PCa cell line.

DAG + etoposide^a^		DAG + camptothecin^b^		DAG + irinotecan^c^		DAG + docetaxel^d^	
Cytotoxic effect (Fa)	Combination index (CI)	Cytotoxic effect (Fa)	Combination index (CI)	Cytotoxic effect (Fa)	Combination index (CI)	Cytotoxic effect (Fa)	Combination index (CI)
ED50	0.58 ± 0.10	ED75	0.68 ± 0.07	ED75	0.78 ± 0.22	ED50	1.40 ± 0.32
ED75	0.48 ± 0.03	ED90	0.59 ± 0.03	ED85	0.59 ± 0.12	ED75	2.11 ± 0.56
ED90	0.42 ± 0.05	ED95	0.54 ± 0.03	ED90	0.49 ± 0.07	ED90	4.60 ± 1.43

^a^Molar ratio of DAG:etoposide is 4.6:1.

^b^Molar ratio of DAG:camptothecin is 250:1.

^c^Molar ratio of DAG:irinotecan is 2.1:1.

^d^Molar ratio of DAG (µM):docetaxel (nM) is 5.1:1 in PC-3 cell line.

**Table 3:** Combination treatments in A549 lung cancer cell line.

DAG + etoposide^a^		DAG + camptothecin^b^	
Cytotoxic effect (Fa)	Combination index (CI)	Cytotoxic effect (Fa)	Combination index (CI)
ED50	0.72 ± 0.14	ED85	0.94 ± 0.16
ED75	0.88 ± 0.04	ED90	0.87 ± 0.15
ED80	0.94 ± 0.08	ED95	0.77 ± 0.14

^a^Molar ratio of DAG:etoposide is 5.1:1.

^b^Molar ratio of DAG:irinotecan is 212:1 in A549 cell line.

Based on our findings that DAG acts in the S phase of cell cycle, we predicted that DAG would have little or no synergistic effect when combined with chemotherapeutic agents that act in the mitotic M phase of cell cycle, such as taxane. The rationale behind this hypothesis was that M phase-targeting drugs will prevent cells from entering S phase required for developing DAG-induced DNA damage and cytotoxicity. Taxane binds to microtubules and prevents these structures from M phase-permissive depolymerization. As such, taxanes are a class of mitotic inhibitors that arrest cell growth at the M phase of cell cycle^55,56^. To test our hypothesis, we performed combination treatments using DAG together with docetaxel in cancer cells, followed by CI value determination. The data confirmed our hypothesis (CI values > 1) of DAG and docetaxel treatment in both M059K and PC-3 cell lines (Tables 1, 2). Combined, these data indicate a potential benefit of combining DAG treatment with S-phase-specific chemotherapeutic drugs, but not with drugs that inhibit progression of cells into S phase.

**Fig. 4:**
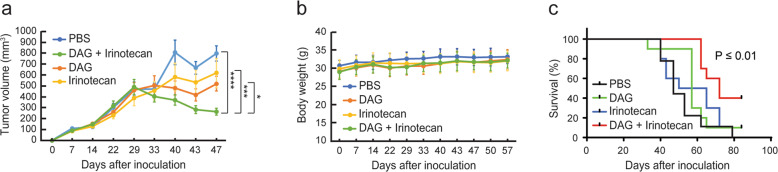
Therapeutic effects of DAG and/or irinotecan in subcutaneous PC-3 tumor model. **a** PC-3 cells were inoculated into the right flank of nude mice and treated with PBS, DAG, irinotecan, or DAG plus irinotecan as described in “Materials and Methods”. Tumor volumes are presented as mean ± standard error of mean. The treatment effectiveness was assessed by comparing the slopes of the tumor grow curves (after day 29) for each group by ANOVA analysis (*p ≤ 0.05; ***p ≤ 0.001; ****p ≤ 0.0001). **b** Body weight monitoring for the four groups of mice in **a**. **c** Kaplan–Meier curve of the survival rates of the four groups of mice in **a**. **p ≤ 0.01.

To evaluate the combined effect in vivo, we implanted PC-3 tumors subcutaneously in nude mice and treated them with DAG and/or irinotecan through intraperitoneal injection. In the attempt to mimic a relevant clinical treatment scenario with alternating single-drug treatments in a combination regimen, the mice were treated with either phosphate-buffered saline (PBS; vehicle), DAG (2.5 mg/kg on days 1, 3, 5 every week for 3 weeks), irinotecan (25 mg/kg on day 4 every week for 6 weeks), or DAG (days 1, 3, 5) in combination with irinotecan (day 4). As compared to treatment with either drug alone, the combination of DAG and irinotecan significantly reduced tumor growth and improved survival in the mice with no signs of toxicity (Fig. 4a–c). These results support the idea that therapeutic efficacy of DAG might be enhanced by combination treatment with topoisomerase poisons in a clinical setting.

## Discussion

DNA inter-strand crosslinks stall cells at replication forks and prevent double-strand separation, eventually leading to DSBs^57^. DAG-induced DSBs trigger replication-dependent S/G_2_ phase cell cycle arrest and activation of the HR DNA damage repair pathway in cancer cells. DNA damage triggers activation of a variety of kinases, including phosphoinositide-3-kinase-related protein kinase family members ATM, ATR, and DNA-PKcs^58^. While ATR kinase is activated by single-stranded DNA-coated RPA protein when DNA replication is impeded, ATM is recruited and responds mainly to DSBs with the association of Mre11-Rad50-Nbs1 (MRN) sensor complex^59^. Activated ATM and ATR kinases trigger orchestration of two major cell cycle checkpoint pathways in cells, leading to activation of Chk2 and Chk1. There are a plethora of local substrates and effectors downstream the ATM kinase, such as p53, H2AX, MRN complex, and Chk2 kinase, collectively involved in controlling cell cycle progression, gene transcription, and apoptosis^60^. Activated ATR triggers phosphorylation of multiple targets, such as RPA32 and Chk1^61^. Also, ionizing irradiation induce ATM-dependent activation of Chk1 on serine 317 and serine 345^62,63^. In this context, we showed that DAG treatment induced activation of both ATM-Chk2 and ATR-Chk1 pathways. The activation of the ATR-Chk1 cascade could be due to stalling of the replication forks exposing single-stranded DNA (ssDNA), or to ssDNA generated by end-resection during the repair of DAG-induced DSBs. Therefore, the ATM-Chk2 and ATR-Chk1-mediated DNA damage signaling pathways could be overlapping under certain circumstances.

The combination of two or more therapeutic agents for cancer treatment is a frequently and successfully used strategy in the clinic. There are essential advantages to combination therapy as compared with conventional mono-therapy. With the synergistic or additive inhibitory effects from combined regimens, the targeting of tumors can be significantly improved^64–66^. In this study, DAG showed synergism with topoisomerase poisons in cancer cells. HR deficiency increased sensitivity of cancer cells to DAG treatment. PARP inhibitors also demonstrate activity in HR-deficient tumors, such as those associated with BRCA mutations^67,68^. Therefore, it might be interesting to further explore a therapeutic strategy of combining DAG with PARP inhibitors in HR-deficient cancers. Meanwhile, a lower dosage of each individual drug in the combination regimen can often be used, which may reduce the risk of adverse effects during and after treatment. In contrast to mono-therapy, combination therapy is less susceptible to drug resistance because of lower dose, fewer treatment cycles, and/or targeting different signaling pathways in cancer patients^64,69–72^. Moreover, combined therapeutics have also demonstrated superiority in eliminating cancer stem cells that play a critical role in disease recurrence^73–75^. A recent study showed DAG’s cytotoxic activity in both TMZ-resistant GBM cells and cancer stem cells^76^. TMZ is a DNA alkylating agent mostly causing N^7^-guanine methylation followed by other targeting sites of O^6^-guanine and O^3^-adenine in DNA^43,77^. The O^6^-methylguanine lesions by TMZ treatment are highly cytotoxic to cells but can be repaired by the MGMT protein present in the majority of GBM tumors, one of the major reasons for TMZ resistance in GBM patients^78,79^. It has previously been shown that DAG overcomes MGMT-mediated resistance in GBM^6^. The present study adds that DAG also seems to circumvent resistance mechanism related to MMR, the secondary TMZ resistance mechanism. Therefore, DAG may prove to be a good therapeutic candidate for TMZ-resistant GBM patients. Moreover, we demonstrated a similar cytotoxic effect and mechanism-of-action of DAG in PC-3 cells, a commonly used model for androgen-independent PCa^80^. As such, our data suggest a potential benefit of using DAG to delay or reverse the progression of PCa, alone or in combination with topoisomerase poisons. In summary, our work describes how GBM and PCa cells respond to DAG treatment and provides a rational for exploring combination regimens with topoisomerase I/II poisons in future clinical trials.

## Materials and methods

### Cell culture and reagents

Human PCa cell lines, PC-3 and DU-145, were cultured in Dulbecco’s Modified Eagle Medium (DMEM, 11995065, Thermo Fisher Scientific, Waltham, MA, USA) supplemented with 10% fetal bovine serum (FBS, Thermo Fisher Scientific). LNCaP cell line was cultured in Rosewell Park Memorial Institute 1640 medium (11875093, Thermo Fisher Scientific) supplemented with 10% FBS. Human GBM cell lines, M059K and M059J, were cultured in DMEM/F-12 medium (11330032, Thermo Fisher Scientific) with 10% FBS. HEK-293T (human epithelia embryonic kidney) cell line was purchased from Takara Bio USA Inc. (632180, Mountain View, CA, USA) and maintained in DMEM plus 10% FBS. Human colorectal carcinoma cell lines, HCT116 and LoVo, were purchased from ATCC (CCL-247 and CCL-229, Manassas, VA, USA). HCT116 was cultured in McCoy’s 5 A medium (30–2007, ATCC) together with 10% FBS. LoVo was maintained in F-12K medium (30–2004, ATCC) plus 10% FBS. A549 cells were also cultured in DMEM supplemented with 10% FBS. Cell lines were maintained in a humidified atmosphere with 5% CO_2_ at 37 °C and routinely tested for mycoplasma. After thawing from liquid nitrogen, cells recovered for two passages before using in the experiments. DAG was provided by DelMar Pharmaceuticals, Inc. (Vancouver, Canada and Menlo Park, CA, USA). Etoposide, irinotecan, camptothecin, and docetaxel were purchased from Selleck Chemicals (Houston, TX, USA). PI solution (1 mg/ml) and glutaraldehyde solution (grade I, 50% in H_2_O) were purchased from MilliporeSigma (Oakville, Canada). Sorenson’s solution was prepared using the following recipe: 9 mg trisodium citrate, 195 ml 0.1 n HCl, 500 ml 90% ethanol and 305 ml distilled water.

### Crystal violet cell proliferation assay

Cells were seeded in 96-well culture plates and incubated at 37 °C overnight. Then the cells were treated with different concentrations of DAG for 72 h followed by fixation in 1% glutaraldehyde solution for 5 min. After the fixation, the cells were washed three times with distilled water and stained with 0.1% crystal violet solution for 10 min. Then, the cells were gently rinsed with distilled water to remove non-stained crystal violet dye from the culture plates. After air drying, the remaining crystal violet dye on the plates was dissolved completely in Sorenson’s solution and absorbance at 560 nm wavelength was measured on a BioTek Gen5 microplate reader. The percentages of survival cells after treatment were determined compared with untreated cells.

### Cell cycle analysis using PI staining

Cells were synchronized by 24 h serum starvation before treatment with 5 μM DAG in complete medium for another 1, 4, 19, 24, 44, and 49 h. After the treatment, cells were trypsinized and centrifuged at 1000 rpm for 5 min. Cell pellets were washed and resuspended in cold PBS followed by fixation in 70% ethanol overnight at 4 °C. After the fixation, the cells were centrifuged at 1500 rpm for 10 min at 4 °C and washed once with cold PBS. Then, cell pellets were resuspended in 500 μl PI solution containing 50 μg/ml PI, 100 μg/ml RNase A, and 0.05% Triton X-100 in PBS and incubated for 40 min at 37 °C in the dark. After that, cells were washed and resuspended in 500 μl cold PBS and filtered with 40 μm cell strainer before DNA content detection using flow cytometry (FACS Canto II). Histograms with cell populations in G_0_/G_1_, S, and G_2_/M cell cycle phases were analyzed using FlowJo software. In parallel, untreated cells were included as control.

### Neutral comet assay

After treatment with or without DAG for indicated conditions, cells were collected and resuspended at 1 × 10^5^ cells/ml in cold PBS (Ca++ and Mg++ free). The neutral comet assay was performed using Trevigen CometAssay kit (4252–040-K, Trevigen, Gaithersburg, MD, USA) following the manufacturer’s protocol. The cells were mixed with molten LMAgarose (at 37 °C) at a ratio of 1:10 (v/v), and 200–400 cells were spread onto each sample area in CometSlide. The slide was kept in the dark for 10 min for gelling followed by overnight incubation in lysis solution at 4 °C. After that, the slide was subjected to electrophoresis in neutral electrophoresis buffer at 25 V for 45 min at 4 °C, followed by incubation in DNA precipitation solution for 30 min at room temperature. Then the slide was in 70% ethanol for 30 min before air drying. SYBR Gold was used to stain the slide for 30 min in the dark. After rinsing with water and air drying, the slide was viewed by Zeiss AxioObserver microscope and ImageJ (OpenComet) software was used for analysis. Tail Moments are presented as mean ± standard deviation.

### Western blot analysis

Cells were lysed with EBC buffer (50 mM Tris-HCl, pH 8.0, 120 mM NaCl, 1% NP-40, and 1 mM ethylenediaminetetraacetic acid; EDTA) supplemented with PhosSTOP phosphatase inhibitor and cOmplete protease inhibitor (MilliporeSigma, Oakville, Canada). Cell lysates were centrifuged at 13,500 rpm for 15 min at 4 °C. Then, protein concentration was determined using Pierce BCA Protein Assay Kit (23225, Thermo Fisher Scientific) following the manufacturer’s instruction. Proteins were separated on 4–15% sodium dodecyl sulfate-polyacrylamide gels (4561084, BioRad Laboratories Ltd., Mississauga, Canada) and transferred onto polyvinylidene fluoride membrane. After blocking for 1 h with 5% bovine serum albumin (BSA) in tris-buffered saline with tween 20 (TBST), the membrane was incubated with corresponding primary antibody overnight at 4 °C. Then, membrane was washed three times with TBST for 10 min each and incubated with horseradish peroxidase-conjugated secondary anti-mouse or anti-rabbit antibodies (Santa Cruz Biotechnology, Dallas, TX, USA) for 1 h at room temperature. After that, membrane was washed three times with TBST for 10 min each and luminescence signal was detected using SuperSignal West Femto Maximum Sensitivity Substrate (34095, Thermo Fisher Scientific) according to the manufacturer’s instruction. The following primary antibodies were used: ɣH2AX (Ser139) (2577, Cell Signaling Technology, Danvers, MA, USA); H2AX (ab11175, Abcam, Toronto, Canada); phospho-ATM (Ser1981) (200–301–400, Rockland Immunochemicals, Inc., Limerick, PA, USA); ATM (2873, Cell Signaling Technology); phospho-RPA32 (Ser33) (A300–246A, Bethyl Laboratories, Montgomery, TX, USA); RPA32 (ab2175, Abcam); phospho-Chk1 (Ser345) (2348, Cell Signaling Technology); phospho-Chk1 (Ser317) (12302, Cell Signaling Technology); phospho-Chk2 (Thr68) (2661, Cell Signaling Technology); BRCA1 (NB100–404, Novus Biologicals, Centennial, CO, USA); MLH1 (4256, Cell Signaling Technology); MSH2 (2017, Cell Signaling Technology); and GAPDH (5174, Cell Signaling Technology). Representative images were shown from three independent experiments.

### IF staining and confocal microscope

Cells were seeded and incubated on glass coverslips in complete medium for 16–20 h in 24-well culture plate. After serum deprivation for 24 h, synchronized cells were treated with or without 50 μM DAG for 1 h followed by washout of the drug and incubation in complete medium for another 24 h. Cells were then washed once with PBS and fixed for 30 min with 4% paraformaldehyde in PBS at room temperature. For confocal microscopic detection of DNA damage foci, cells were pre-incubated with cytoskeletal buffer for 5 min at 4 °C following the recipe: 25 mM HEPES, pH 7.4, 50 mM NaCl, 1 mM EDTA, 3 mM MgCl_2_, 300 mM sucrose, and 0.5% Triton X-100. Then, cells were fixed with 4% paraformaldehyde in PBS for 30 min at room temperature. After fixation, cells were washed three times with PBS for 5 min each and permeabilized with 0.5% Triton X-100 in PBS for 20 min. Following another three times wash with PBS and blocking with 3% BSA in PBS for 1 h at room temperature, cells were incubated with designated primary antibody overnight at 4 °C. Then, cells were washed three times with PBS for 5 min each and incubated with fluorophore-labeled secondary antibody for 1 h at room temperature. After washing with PBS for another three times, the coverslips with cells were mounted with Vectashield mounting medium (with 4’,6-diamidino-2-phenylindole). Images were captured by Zeiss AxioObserver microscope and confocal LSM-780 microscope and analyzed by LSM-ZEN software. The following antibodies were used: ɣH2AX (2577, Cell Signaling Technology); ɣH2AX (05–636, MilliporeSigma, Oakville, Canada); cyclin A2 (ab16726, Abcam); BRCA1 (ab16780, Abcam); Rad51 (H8349, Santa Cruz Biotechnology); RPA32 (ab2175, Abcam); donkey anti-rabbit Alexa-Fluor 594 (A21207, Thermo Fisher Scientific); donkey anti-rabbit Alexa-Fluor 488 (A21206, Thermo Fisher Scientific); donkey anti-mouse Alexa-Fluor 594 (A21203, Thermo Fisher Scientific); and donkey anti-mouse Alexa-Fluor 488 (A21202, Thermo Fisher Scientific). Representative IF and confocal microscopic images were from three independent experiments.

### Transient transfection with siRNAs

PC-3 or M059K cells were transfected with control siRNA or siRNAs targeting BRCA1 using Lipofectamine RNAiMAX Transfection Reagent (13778075, Thermo Fisher Scientific) following the manufacturer’s protocol. After 24 h transfection, cells were trypsinized and seeded in 96-well plates in complete medium. The next day, transfected cells were treated with different concentrations of DAG for 5 days and followed by crystal violet assay as described before. Cell lysates were collected and protein extraction was analyzed by western blot to confirm BRCA1 knockdown. The siRNAs used were as follows: C, siCon (462001, negative control medium GC duplex, Invitrogen); B1, siBRCA1–2 (SI00096313, Qiagen, Toronto, Canada); B2, siBRCA1–14 (SI02664361, Qiagen); B3, siBRCA1–25 (SI04381377, Qiagen); B4, siBRCA1–15 (SI02664368, Qiagen); and B5, siBRCA1–17 (SI03103975, Qiagen).

### Stable cell line generation

Human MLH1 cDNA (obtained from vector RC201607, OriGene Technologies, Rockville, MD, USA) or MSH2 cDNA (obtained from vector RC205848, OriGene Technologies) was subcloned into pLenti-C-Myc-DDK-P2A-Puro vector (PS100092, OriGene Technologies) using SgfI and MluI restriction enzyme sites to construct PS-MLH1 or PS-MSH2 expression vector. Isogenic HCT116 cell line expressing MLH1 (HCT116-PS-MLH1) or LoVo cell line expressing MSH2 (LoVo-PS-MSH2) was generated by lentivirus transduction of the construct carrying corresponding gene (PS-MLH1 or PS-MSH2). PS100092 was included as negative control. Plasmids pMD2.G and psPAX2 were gifts from Didier Trono (12259 and 12260, Addgene, Watertown, MA, USA) and were used for lentivirus particle packaging in HEK-293T cells. After lentivirus transduction, HCT116-PS-MLH1 and LoVo-PS-MSH2 stable cell lines were selected using 0.3 µg/ml and 4 µg/ml puromycin (ant-pr-1, InvivoGen, San Diego, CA, USA) in complete medium, respectively.

### Combination treatment and CI determination

The cytotoxic effects of DAG, etoposide, irinotecan, camptothecin, and docetaxel on cell survival were determined by crystal violate assay as described before. The IC_50_ values of each drug for 72 h treatment were determined using GraphPad Prism 6.0 software. After that, cells were exposed to combination treatment with fixed concentration ratios of different drugs based on their corresponding IC_50_ values (In PC-3 cells, DAG: etoposide = 4.6:1, DAG: camptothecin = 250:1, DAG: irinotecan = 2.1:1, DAG: docetaxel = 5.1:1; In M059K cells, DAG: etoposide = 2.5:1, DAG: irinotecan = 3.4:1, DAG: docetaxel = 1.3:1; In A549 cells, DAG: etoposide = 5.1:1, DAG: camptothecin = 212:1). Different combinations (e.g., ranging from one-tenth of the IC_50_ concentration to 10 times of the IC_50_ of each drug) were tested in three to four independent experiments with triplicate samples. The CI values for each combination were calculated according to Chou–Talalay method^54^ with Calculsyn software (Biosoft, Version 2.0) to quantitatively determine the nature of two-drug interactions (CI < 1, synergism; CI = 1, additivity; CI > 1, antagonism).

### Tumor implantation and treatment in mice

Male nude (*Nu/Nu*) mice (6–7 weeks old, Jackson Laboratory) were blindly allocated into different cages and maintained in standard pathogen-free conditions. All the experimental procedures were carried out in accordance with protocols, ethical regulations and guidelines (A20–0046) approved by the Institutional Animal Care Committee (IACC) at the University of British Columbia. The mice were injected subcutaneously in the right flank with 100 μl PC-3 cell suspension (1 × 10^6^ cells in 50% PBS and 50% Matrigel; Corning, 354234, Tewksbury, MA, USA). The tumor volumes were monitored every week by caliper measurement using formula *V* = *π*(length × width × height)/6. When the tumor size reached 100–200 mm^3^, the mice were randomly divided into four groups (9–10 mice/group) and treated with intraperitoneal injections of PBS (vehicle), DAG (2.5 mg/kg on days 1, 3, 5 every week for 3 weeks), irinotecan (25 mg/kg on day 4 every week for 6 weeks), or DAG in combination with irinotecan. Tumor growth was monitored using caliper measurement and the mice were sacrificed when the tumors reached humane endpoint (900–1000 mm^3^ or ulceration).

### Statistical analysis

Data were presented as mean ± standard deviation from three to four independent experiments. Where indicated, Student’s *t* tests were performed to calculate *p* values for statistical significance.

## Supplementary information

Supplementary figure

Supplementary figure legends
