# Supplementary figures and images for: Dianhydrogalactitol synergizes with topoisomerase poisons to overcome DNA repair activity in tumor cells
Source: Cell Death Dis. 2020 Jul 24;11(7):577. doi: 10.1038/s41419-020-02780-8 (PMC7381652; doi:10.1038/s41419-020-02780-8)

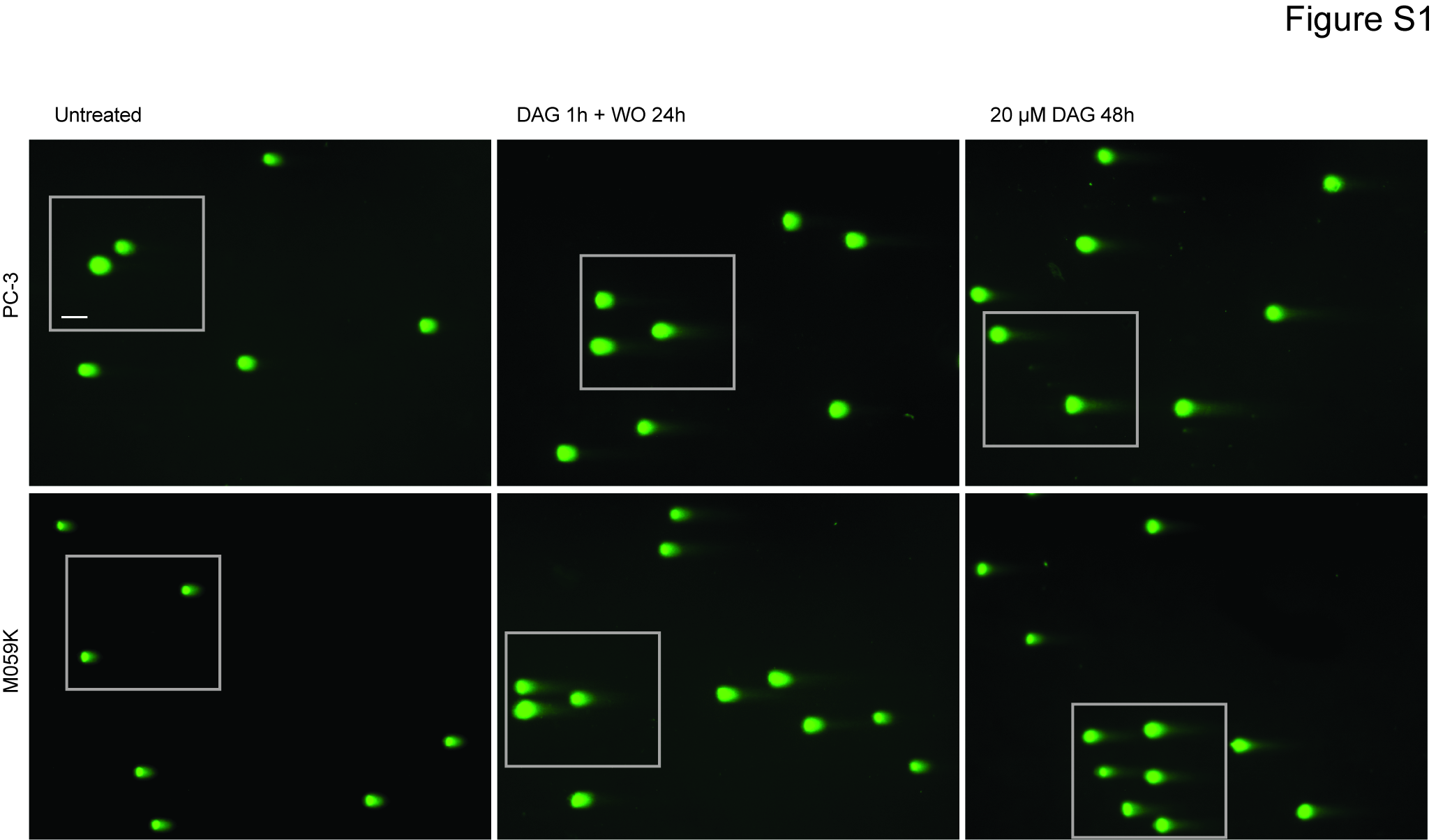

Supplement: Supplementary file 1 — Supplementary figure [file 41419_2020_2780_MOESM1_ESM.tif]
