# Supplementary material for: Dianhydrogalactitol synergizes with topoisomerase poisons to overcome DNA repair activity in tumor cells
Source: Cell Death Dis. 2020 Jul 24;11(7):577. doi: 10.1038/s41419-020-02780-8 (PMC7381652; doi:10.1038/s41419-020-02780-8)
Supplement: Supplementary file 2 — Supplementary figure legends [file 41419_2020_2780_MOESM2_ESM.docx]

**Supplementary Figure Legends:**

**Figure S1:** Uncropped microscopic images related to **Fig. 2e**. PC-3 and M059K cells were subjected to neutral comet assay with or without DAG treatment. The scale bar stands for 50 µm.
